# Self-admission in the treatment of eating disorders: an analysis of healthcare resource reallocation

**DOI:** 10.1186/s12913-021-06478-1

**Published:** 2021-05-17

**Authors:** Mattias Strand, Cynthia M. Bulik, Sanna A. Gustafsson, Elisabeth Welch

**Affiliations:** 1 Centre for Psychiatry Research, Department of Clinical Neuroscience, Karolinska Institutet, & Stockholm Health Care Services, 171 77 Stockholm, Sweden; 2 Transkulturellt Centrum, Solnavägen 4, 113 65 Stockholm, Sweden; 3 Department of Medical Epidemiology and Biostatistics, Karolinska Institutet, Stockholm, Sweden; 4 Department of Psychiatry, University of North Carolina at Chapel Hill, Chapel Hill, North Carolina USA; 5 Department of Nutrition, University of North Carolina at Chapel Hill, Chapel Hill, North Carolina USA; 6 University Health Care Research Center, Faculty of Medicine and Health, Örebro University, 701 82 Örebro, Sweden; 7 Stockholm Centre for Eating Disorders, Stockholm Health Care Services, Stockholm County Council, Stockholm, Sweden

**Keywords:** Economics, Delivery of health care, Health care rationing, Health resources, Voluntary admission, Anorexia nervosa

## Abstract

**Background:**

Self-admission to psychiatric inpatient treatment is an innovative approach to healthcare rationing, based on reallocation of existing resources rather than on increased funding. In self-admission, patients with a history of high healthcare utilization are invited to decide for themselves when brief admission is warranted. Previous findings on patients with severe eating disorders indicate that self-admission reduces participants’ need for inpatient treatment, but that it does not alone lead to symptom remission.

**Methods:**

The aim of this study was to evaluate if, from a service provider perspective, the resource reallocation associated with self-admission is justified. The analysis makes use of data from a cohort study evaluating the one-year outcomes of self-admission at the Stockholm Centre for Eating Disorders.

**Results:**

Participants in the program reduced their need for regular specialist inpatient treatment by 67%. Thereby, hospital beds were made available for non-participants due to the removal of a yearly average of 13.2 high-utilizers from the regular waiting list. A sensitivity analysis showed that this “win-win situation” occurred within the entire 95% confidence interval of the inpatient treatment utilization reduction.

**Conclusions:**

For healthcare systems relying on rationing by waiting list, self-admission has the potential to reduce the need for hospitalization for patients with longstanding eating disorders, while also offering benefits in the form of increased available resources for other patients requiring hospitalization.

**Trial Registration:**

ClinicalTrials.gov ID: NCT02937259 (retrospectively registered 10/15/2016).

## Background

Self-admission to psychiatric inpatient treatment is an innovative collaborative approach to healthcare rationing in which patients with a history of high utilization of inpatient treatment are offered the choice to decide for themselves when a brief hospital admission episode—usually 3–7 days at a time—is warranted. Self-admission has been used in Norwegian and Dutch psychiatry for over a decade as a tool in the treatment of enduring mental illness such as schizophrenia, bipolar disorder, and borderline personality disorder [1]. Similar programs have more recently been introduced in Sweden [2–4] and Denmark [5].

 Participants self-admit by contacting the designated ward directly. Central to the approach is that the patients’ reasons for choosing to self-admit (such as deteriorating mental health, acute crisis, poor everyday structure, loneliness, or any other reason) are not questioned. Hence, the traditional inpatient admission model with a clinician operating as gatekeeper is bypassed. Participants are also free to discharge at will. Self-admission programs function by giving high utilizers of healthcare a “fast lane” to admission, without waiting time and risk of being sent home by a psychiatric emergency service. Participants typically have a history of multiple and prolonged episodes of inpatient treatment. By encouraging self-monitoring of their mental health status and promoting early help-seeking, the delay between the first signs of deterioration and hospital admission can hopefully be minimized. This may in turn reduce the need for prolonged episodes of inpatient treatment—if patients are invited to act early on subjective cues that might not prompt admission if assessed through a regular emergency service, they can potentially avoid further deterioration and lengthier hospital admissions.

The self-admission program at the Stockholm Centre for Eating Disorders (SCÄ) evaluated in the present paper is the first to target patients with longstanding eating disorders, mainly those suffering from anorexia nervosa (AN). AN is characterized by restriction of energy intake resulting in a significantly low body weight, an intense fear of weight gain, and a disturbed experience of one’s own body weight or shape [6]. Treatment for AN can often be successfully administered in outpatient settings [7, 8]. Still, a prolonged trajectory with enduring disability is seen in as many as 20–30 % of individuals with AN [9, 10]. Some patients require lengthy periods of inpatient treatment and relapse after discharge is common—a scenario that may evolve into a “revolving door” pattern [11].

**Fig. 1:**
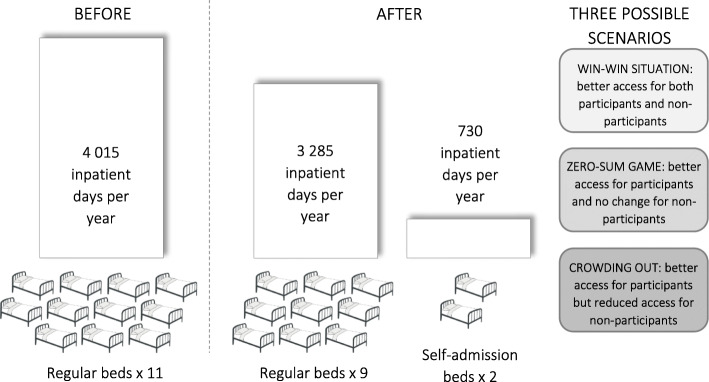
Resource reallocation in the self-admission program at the Stockholm Centre for Eating Disorders

SCÄ is a public sector specialist service for the treatment of eating disorders in Stockholm, Sweden operared by the Stockholm County Council. The catchment area is Metropolitan Stockholm with a population of 2.2 million. Treatment at the hospital is publicly funded, with only minor patient fees in consonance with all Swedish public healthcare. The self-admission program at SCÄ has previously been described in detail elsewhere [3, 12]. In brief, two beds out of eleven at the adult inpatient ward are earmarked for patients in the self-admission program (see Fig. 1). Because of the typically prolonged nature of regular admissions, the patient turnover at the ward is low and there is usually a several weeks wait for regular admission. Emergency admissions are not available. In contrast, participants in the self-admission program can admit themselves at will as often as they prefer for a maximum of 7 days at a time by contacting the ward directly. A waiting list is established whenever both designated beds are already occupied by other program participants. Self-admission is designed as an add-on tool and admission through regular procedures is still available if the situation warrants. Eligible program participants must maintain outpatient or day treatment contact at the clinic, they must have been admitted for specialized inpatient treatment at least once in the previous three years, and they must have the ability to follow the basic treatment framework at the ward. Typically, inclusion in the program is suggested by a patient’s treatment contact at the clinic and the functions of model are discussed in-depth before the patient decides whether to join.

In a qualitative study, participants in the self-admission program at SCÄ reported a high level of satisfaction, increased agency, and strengthened motivation [12]. Moreover, in terms of quantitative outcomes, a 51 % reduction in time spent hospitalized at one-year follow-up (at SCÄ and all other Swedish health services) was seen among participants, compared to non-significant changes among comparison groups [13]. In contrast, participants’ body mass index (BMI) or eating disorder morbidity were not affected during their time in the self-admission program. In terms of health-related quality of life (HRQoL), inconclusive outcomes were found. Regarding sick leave, a beneficial but statistically non-significant pattern was seen for participants. Taken together, these results show that self-admission is a viable and useful tool primarily for reducing participants’ need for inpatient treatment by promoting swift help seeking, but alone it does not lead to symptom remission in patients with longstanding illness.

From a health economic perspective, the self-admission approach is based on reallocation of existing healthcare resources (i.e., hospital beds) rather than on increased funding and capacity expansion. Since the beds designated for self-admission are taken from the pool of regular beds, the number of beds available for those patients who are not included in the self-admission program is inevitably reduced. However, if those patients who are removed from the regular hospital queue and offered a separate “fast lane” to brief admission are able to use this tool in a way that reduces the total time they spend hospitalized, the regular queue may be shortened to the extent that it balances out the loss of the reallocated hospital beds [3].

Three scenarios could arise when self-admission is introduced (see Fig. 1). First, having lost healthcare resources that were previously available to them, the non-participants could be subjected to crowing out and end up in a worse off situation than before. Second, a “zero-sum game” can be imagined, whereby the benefits accrued by participants do not have negative impact on availability for non-participants. Third, a “win-win situation” could hypothetically be created, in which increased access and better outcomes for participants means that hospital beds are freed up to be used by non-participants [14].

It should be noted that hospital beds reserved for participants in the self-admission program are not meant to be occupied all of the time, the way regular hospital beds would ideally be. This is because of the “safety net” function of self-admission: if participants could not count on these beds to be available at need and thus largely unoccupied, a core element of the self-admission program would be lost. Justifying the need for keeping expensive and sought-after hospital beds unoccupied may, however, be challenging from a service provider perspective.

### Objective

The aim of the present analysis is to evaluate if, from a healthcare service provider perspective, the reallocation of resources associated with the introduction of self-admission is justified, such that the reduction of hospital beds available for regular patients on the waiting list is offset by the observed reduction in healthcare utilization for the program participants.

## Methods

### Study design and target population

The present analysis makes use of data from a cohort study evaluating the one-year outcomes of self-admission at SCÄ in terms of healthcare utilization, eating disorder morbidity, HRQoL, and sick leave. All patients who partook in the SCÄ self-admission program at some point between August 2014 and February 2019 were invited to also participate in the study. All of them agreed to have their data on healthcare utilization collected. In total, 29 participants (27 women and two men) in the self-admission program, all with an AN diagnosis, were followed for one year. Participants’ mean age was 29.7 years, their mean duration of illness was 13.4 years, and their mean BMI was 15.8 kg/m^2^ (i.e., severe underweight). During the study period, they met with a researcher briefly on three occasions to respond to questionnaires etc.; thus, the healthcare provided to participants in the study did not differ significantly from routine practice except for their access to self-admission. Using Stepwise, a nationwide eating disorder database that has been found to be valid and reliable [15, 16], a comparison group matched on age, duration of illness, and BMI was identified. Data on patients in treatment at specialist eating disorder services throughout Sweden were routinely entered into Stepwise during the years covered by this study, and individuals from this source thus represent a historical “treatment as usual” (TAU) population. An overall 1:4 ratio of participants to comparison group patients was achieved, although for two participants a lower ratio had to be accepted due to a scarcity of patients with matching severity of illness in Stepwise. In total, the comparison group comprised 113 patients whose data were entered into the Stepwise register between 2013 and 2017 [13].

### Outcome measures

For analyzing changes in healthcare utilization, data from the nationwide registers maintained by the Swedish government, which covers the Swedish population in its entirety [17], were used. Specifically, data on the number of days and frequency of inpatient treatment during one year prior to and after baseline were retrieved from the National Patient Register, kept by the Swedish National Board of Health and Welfare. Data on participants’ self-admission episodes and number of participants in the program were retrieved from patient records. In sum, the cohort study on which the present analysis is based showed a statistically significant (*p* = .001) 51.1 % reduction in time spent hospitalized (at SCÄ and all other Swedish health services) at one-year follow-up, compared to a statistically non-significant (*p* = .259) 34.7 % increase in the TAU comparison group [13]. However, participants’ BMI or eating disorder morbidity was not affected in a clinically meaningful way during follow-up and HRQoL outcomes were inconclusive. Typically, a cost-effectiveness analysis is based on the assumption that an intervention leads to specific measurable health consequences that can then be related to the costs associated with the intervention. Here, however, the main outcome of the self-admission intervention was a reduction of time spent in inpatient treatment, whereas eating disorder symptoms and HRQoL were largely unaffected. Thus, they have not been included as parameters in the present economic evaluation. Since this evaluation concerns the reallocation of resources at SCÄ only, all healthcare utilization data from the original cohort study have been recalculated so as to exclude other Swedish healthcare services.

### Costs

In the purchaser-provider model employed by the Stockholm County Council, regular admission episodes and self-admission episodes are both remunerated in the same manner, as fixed sums based on full bed occupancy.

Start-up costs in the program mainly included staff education and the printing of patient information material. As self-admission is based on reallocation of existing resources, no additional inventorial costs were accrued. Experiences from SCÄ [18] shows that it was fully possible to incorporate these activities into regular staff meetings and continuous education. Likewise, the printing of patient information folders represented a very minor cost that was accommodated within the regular hospital budget for patient education materials. Overall, the start-up costs in a self-admission program are negligible and have not been included in the present analysis.

Taken together, while there are no direct costs associated with the self-admission program—the fixed healthcare service budget remains the same and no resources (financial or other) are added—there are opportunity costs from displaced activities in the form of 730 inpatient days per year (2 beds x 365 days) that are no longer available for regular patients at SCÄ (see Fig. 1), regardless of how many of these inpatient days that are in fact instead utilized by program participants.

### Analysis

The analysis is primarily concerned with whether the opportunity cost mentioned above is offset by a large enough reduction in the number of days that program participants spend in inpatient treatment. Although limited in scope, this approach is somewhat similar to a distributional cost-effectiveness analysis in that it takes into account equity concerns related to the opportunity cost of displaced activities within a fixed healthcare service budget [19].

In the reallocation of resources associated with self-admission, opportunity costs and benefits (i.e., reduction in participants’ time spent hospitalized) are generated over the same time horizon—in this particular study, over the course of one year. Accordingly, no discounting has been applied in the analysis.

In the cohort study that this analysis is based on, Wilcoxon signed-rank tests were performed for changes in healthcare utilization due to the fact that the differences between pairs were generally not normally distributed. This notwithstanding, for the present study paired t-tests were performed in order to calculate a 95 % confidence interval (CI) for sensitivity analysis purposes (see below). An *α* level of < 0.05 was considered statistically significant. Performing Wilcoxon signed-rank tests did not alter the statistical significance of the findings. IBM® SPSS® Statistics 26 was used for all statistical analyses.

### Sensitivity analysis

To account for stochastic parameter uncertainty, 95 % CIs for the healthcare utilization estimates were used. Moreover, threshold values regarding number of participants and reduction of the number of inpatient days were calculated.

### Ethics, pre-registration, and adherence to reporting guidelines

The study was approved by the Swedish Ethical Review Authority (Nos. 2014/1586-31, 2015/1537-32, 2018/1184-32, and 2020 − 00831). The study protocol was pre-registered at ClinicalTrials.gov (ID: NCT02937259). In reporting our findings, we have adhered to the Consolidated Health Economic Evaluation Reporting Standards (CHEERS) statement [20].

## Results

### Main analysis

**Fig. 2:**
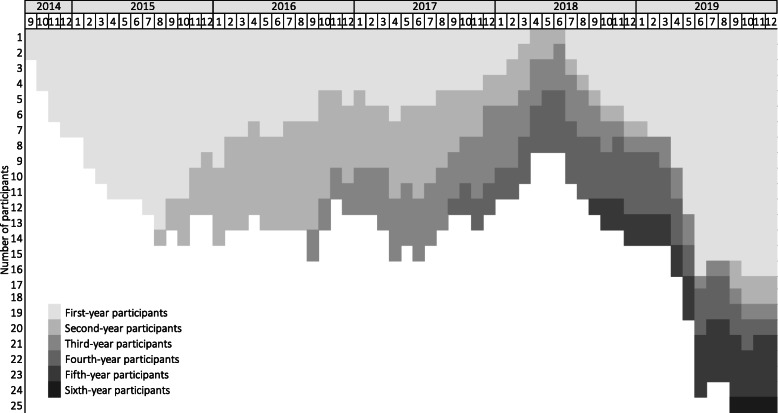
Participants over time in the self-admission program at the Stockholm Centre for Eating Disorders

Patterns of utilization of self-admission over time in the program are shown in Fig. 2. Over the entire study period, 41 patients (including those who had not yet been in the program for one year at the end of the study) were enrolled in the program. The mean number of program participants at any given time since the launch in August 2014 until December 2019 was 13.2. Since participants were included consecutively, there was a gradual increase in the number of participants in the first year, followed by a stabilization. The increase during the last part of the observation period was most likely due to the fact that several original participants remained in the program for “safety net” reasons even though they did not actively make use of the self-admission option, which allowed for the further inclusion of new participants.

**Fig. 3:**
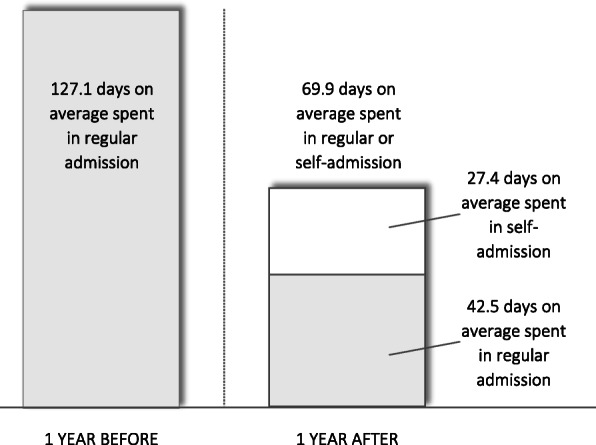
Change in the mean number of days spent in inpatient treatment at the Stockholm Centre for Eating Disorders

**Fig. 4:**
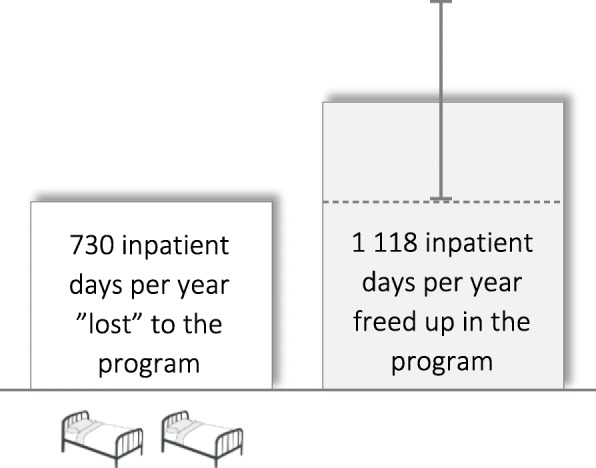
Net increase in available regular hospital days outnumbering the days “lost” to the program

The mean number of regular inpatient days at SCÄ for participants during the one year before and after inclusion were 127.1 and 42.5, respectively, corresponding to a 66.6 % reduction in regular inpatient days at SCÄ specifically (excluding utilization of other healthcare services; see Fig. 3). Thus, on average, the utilization of regular inpatient treatment at SCÄ for participants was reduced by 84.7 days (95 % CI: 55.7-113.7; *p* < .001). Assuming a yearly average of 13.2 participants, this resulted in a net increase of available regular inpatient days of 1118.0, outnumbering the available regular hospital days “lost” to the program (i.e., 730 days; see Fig. 4) by 53.2 %. Therefore, a clear “win-win situation” was observed.

The mean total number of inpatient days at SCÄ (including both regular and self-admission) for participants during the one year after inclusion was 69.9, corresponding to a 44.1 % reduction (see Fig. 3). Thus, on average, the total utilization of inpatient treatment at SCÄ for participants was reduced by 57.3 days (95 % CI: 21.7–92.9; *p* = .003). The mean number of self-admission days during the one year after inclusion was 27.4. Assuming a yearly average of 13.2 participants, the earmarked beds were actively utilized during 361.7 self-admission days out of the available 730. Thus, the earmarked beds were utilized 49.5 % of the time available.

### Sensitivity analysis

Utilizing the low end of the 95 % CI—i.e., a reduction of the utilization of regular inpatient treatment at SCÄ for participants by 55.7 days—for a sensitivity analysis resulted in an increase of available regular inpatient days of 735.2, i.e., just above the 730 days “lost” to the program annually. Utilizing the high end of the 95 % CI—i.e., a reduction of the utilization of regular inpatient treatment at SCÄ for participants by 113.7 days— resulted in an increase of available regular inpatient days of 1500.8, exceeding the number of available regular hospital days “lost” to the program by 105.6 %. Thus, across the entire 95 % CI, the self-admission program resulted in a “win-win situation” (see Fig. 3).

Threshold analyses showed that given the present findings in terms of reduction of days spent in regular inpatient treatment, break-even occurred at nine participants (where a reduction of a total 762.2 days was displayed). Likewise, given an average number of 13 participants, break-even occurred at a reduction of days spent in regular inpatient treatment of 56.2 days per participant annually.

## Discussion

This study shows that by establishing a program of self-admission to inpatient treatment for patients with eating disorders, a “win-win situation” in terms of resource allocation was achieved. In the program, participants with longstanding illness were offered access to earmarked hospital beds and were able to reduce their utilization of regular inpatient treatment at SCÄ by 67 %. In doing so, hospital beds were made available for non-participants due to the removal of a yearly average of 13.2 high-utilizers of inpatient treatment from the regular waiting list. A sensitivity analysis showed that this “win-win situation” occurred across the entire 95 % CI of the inpatient treatment utilization reduction.

Available findings indicate that being offered self-admission as a tool in the treatment of eating disorders helped participants prevent deterioration and reduce their need for inpatient treatment, but that it did not aid in achieving symptom remission such as a normalized weight or reduced eating disorder cognitions [13]. Considering that the targeted participant group displayed a mean duration of illness of 13.4 years, self-admission certainly should not be expected to provide a “quick fix”. Instead, these brief admissions are best thought of as booster opportunities or brief respites at times when the risk of deterioration is high. As we have discussed in more detail elsewhere [1, 13, 21], self-admission is therefore probably best understood within a recovery model framework. In this model, a distinction is made between recovery *from* and recovery *in* a disorder [22]. In recovery *from* a disorder, the focus is on a traditional notion of cure. In contrast, recovery *in* a disorder implies that even though patients may still fulfill diagnostic criteria, they have access to tools that help them manage symptoms and increase their quality of life despite not being formally cured. There are numerous examples of treatment approaches incorporating aspects of the recovery model, harm reduction principles, etc. for patients with AN [23–28]. For the present study, proper cost-effectiveness or cost-utility analyses were not feasible due to the fact that eating disorder morbidity and HRQoL were ultimately unaffected by participation in the program. Even so, the substantial observed reduction in the need for hospitalization should be considered a highly positive and clinically relevant outcome within a recovery model framework for patients with severe and longstanding illness. It can be noted that in previous qualitative research, a main finding was that access to self-admission helped participants expand their scope of everyday activities and increase their overall quality of life [12], although corresponding improvements in HRQoL were not observed when assessed with commonly used self-rating instruments [13]. For a discussion of why standard HRQoL self-rating instruments may in fact not be optimal for the assessment of individuals with longstanding AN (such as, for instance, a tendency for negative response shift after entering treatment in this group), see Strand et al. 2020 [13].

The two hospital beds earmarked for the self-admission program were on average utilized 49.5 % of the time. Here, it should be remembered that the perceived—and real—availability of free hospital beds is a prerequisite for the program to function and that since a reduced need for inpatient treatment is a core aim of the intervention, a low hospital bed utilization within the program is in itself a positive outcome. The present utilization rate is actually comparatively high considering previous studies, where the hospital beds reserved for self-admission have been occupied for 24–31 % [1]. Thus, perhaps somewhat counterintuitively, the discussion about health services efficiency with regards to self-admission is fundamentally not about whether the designated hospital beds should primarily be offered to regular patients or solely to those who participate in the program, but rather about whether they should be offered to regular patients or largely remain empty and ready for use by participants once they need them. However, noting that the SCÄ hospital beds were “only” utilized just under half of the time available, it may be tempting for healthcare providers to simply reassign one of the two beds to the pool of regular patients and thereby seemingly increase the “win-win” efficiency of the program even further. Considering that the perceived availability of beds may hypothetically alter the overall equilibrium of the program (e.g., making participants more eager to self-admit if they have the impression that the designated beds are too scarce), such a reallocation of resources may not have the intended result. In future self-admission programs, the optimal proportion of hospital beds assigned to self-admission at a ward should be carefully followed.

On a similar note, even though threshold values for number of participants and reduction of the number of inpatient days were calculated for the purpose of sensitivity analysis, the number of participants in the program or the proportion of hospital beds earmarked for them are intimately related to the outcome in terms of reduced healthcare utilization in a complex, reciprocal manner that makes a straightforward analysis difficult. Clearly, the findings presented here do not imply that one can simply increase the number of earmarked beds or program participants and achieve a corresponding increase in inpatient days made available for non-participants—at a certain point, an equilibrium is reached.

### Strengths and limitations

Even though self-admission has now been offered in several countries for over a decade, this is the first health economic evaluation of the model. This study relies on data from well-established high quality Swedish population registers [17, 29]. Likewise, the methods for data collection for the nationwide eating disorder database Stepwise have been found to be valid and reliable [15, 16].

Still, the findings reported here should be considered in the light of several limitations. First, it may not be feasible to introduce a service delivery model characterized by patient choice and high levels of flexibility in all settings, since there is a large variance between countries in how healthcare systems operate [3, 11]. For example, the present study relies on rationing by waiting list, whereas some healthcare systems practice rationing by ability to pay. This limits the transferability of our findings.

Second, even though the self-admission program could be seen as an experimental approach per se, it was not feasible to carry out a formal experimental study of the program. Early on, the Stockholm County Council decided that self-admission should be made widely available and rolled out on a broader scale, although there was not yet sufficient evidence to suggest that the model was effective. This decision meant that it would not have been possible to randomly allocate eligible patients to different study arms—i.e., active participation in the self-admission program or a control condition such as TAU—since this would have involved offering patients treatment on unequal terms, which is not usually seen as acceptable once a treatment intervention has been established and confirmed by government agencies as the treatment of choice and a standard option. Instead, the intervention had to be evaluated using a cohort study approach. Ideally, an economic evaluation of a novel healthcare intervention should be based on data from a randomized controlled trial. Even so, data from observational studies can provide robust evidence on cost-effectiveness, not least in situations when a policy initiative is introduced in a way that makes experimental research designs less feasible [30]. Unfortunately, once an intervention is formally approved and established, the likelihood of further experimental research being conducted is reduced, which means that decision makers should also take account of the value of the evidence that is forgone when a policy is hastily introduced [31].

Third, it should be noted that by applying a strict healthcare service provider perspective, the present analysis does not take patient opportunity costs (e.g., lost income during admission or travel costs) or broader non-health sector benefits (e.g., being able to return to work or school faster because of a reduced need of inpatient treatment) into account. Data indicate that there may indeed be additional benefits in terms of reduced days in sick leave for participants [13], although this tendency was not statistically significant. Moreover, as a basis for healthcare sector decision making, broader aspects related to patient satisfaction [12] and equity [14] need to be carefully considered. For example, it may be argued that self-admission prioritizes the worse-off patients with longstanding illness; on the other hand, all patients with AN that require inpatient treatment are by definition suffering from a severe life-threatening disorder [32].

Furthermore, the present resource reallocation analysis only takes eating disorder specialist treatment at SCÄ into account. In Stockholm County, an individual can in practice only maintain one active eating disorder specialist treatment contact at once. Therefore, it is certain that participants did not partake in parallel eating disorder specialist treatment at another clinic during follow-up. However, many of them had active treatment contacts in general psychiatry, as well as in primary care and somatic medicine. As shown in the original cohort study, participants reduced their overall need for inpatient treatment (including other healthcare services) by 51.1 % [13], even though the reduction of regular inpatient days at SCÄ specifically observed in the present study, at 66.6 %, was even greater. Thus, the resource reallocation at SCÄ led to positive ripple effects freeing up resources in other branches of the healthcare sector too, which have not been accounted for here.

## Conclusions

This is the first health economic evaluation of the self-admission model in psychiatry. In sum, the findings show that by establishing a program of self-admission to inpatient treatment for patients with eating disorders, a “win-win situation” in terms of resource allocation was achieved, so that the reduction of hospital beds available for regular patients on the waiting list was offset by an even larger reduction in healthcare utilization for program participants. The two hospital beds earmarked for self-admission were on average utilized 49.5 % of the time, reflecting a need for surplus capacity within the system in order for the model to function effectively. For healthcare systems relying on rationing by waiting list, self-admission has the potential to transform healthcare from crisis-driven to pre-emptive and reduce the need for hospitalization for patients with longstanding eating disorders, while also offering benefits in the form of increased available resources for non-participants.

## Data Availability

The datasets used and analysed during the current study are available from the corresponding author on reasonable request.

## Abbreviations

AN: Anorexia nervosa
BMI: Body mass index
CI: Confidence interval
HRQoL: Health-related quality of life
SCÄ: Stockholm Centre for Eating Disorders
TAU: Treatment as usual

## References

[1] Strand M, von Hausswolff-Juhlin Y (2015). Patient-controlled hospital admission in psychiatry: A systematic review. Nord J Psychiatr.

[2] Eckerström J, Allenius E, Helleman M, Flyckt L, Perseius K-I, Omerov P (2019). Brief admission (BA) for patients with emotional instability and self-harm: nurses’ perspectives - person-centred care in clinical practice. Int J Qual Stud Health Well-being.

[3] Strand M, Gustafsson SA, Bulik CM, von Hausswolff-Juhlin Y (2015). Patient-controlled hospital admission: A novel concept in the treatment of severe eating disorders. Int J Eat Disord.

[4] Westling S, Daukantaitė D, Liljedahl SI, Oh Y, Westrin Å, Flyckt L (2019). Effect of brief admission to hospital by self-referral for individuals who self-harm and are at risk of suicide: a randomized clinical trial. JAMA Netw Open.

[5] Thomsen CT, Benros ME, Maltesen T, Hastrup LH, Andersen PK, Giacco D (2018). Patient-controlled hospital admission for patients with severe mental disorders: a nationwide prospective multicentre study. Acta Psychiatr Scand.

[6] American Psychiatric Association. DSM-5: Diagnostic and Statistical Manual of Mental Illness, Fifth Edition. Washington, DC: American Psychiatry Publishing; 2013.

[7] Brockmeyer T, Friederich H-C, Schmidt U (2018). Advances in the treatment of anorexia nervosa: a review of established and emerging interventions. Psychol Med.

[8] Keel PK, Brown TA (2010). Update on course and outcome in eating disorders. Int J Eat Disord.

[9] Dobrescu SR, Dinkler L, Gillberg C, Råstam M, Gillberg C, Wentz E (2020). Anorexia nervosa: 30-year outcome. Br J Psychiatr.

[10] Eddy KT, Tabri N, Thomas JJ, Murray HB, Keshaviah A, Hastings E (2017). Recovery From Anorexia Nervosa and Bulimia Nervosa at 22-Year Follow-Up. J Clin Psychiatr.

[11] Wonderlich SA, Bulik CM, Schmidt U, Steiger H, Hoek HW (2020). Severe and enduring anorexia nervosa: Update and observations about the current clinical reality. Int J Eat Disord.

[12] Strand M, Bulik CM, von Hausswolff-Juhlin Y, Gustafsson SA (2017). Self-admission to inpatient treatment for patients with anorexia nervosa: the patient’s perspective. Int J Eat Disord.

[13] Strand M, Bulik CM, Gustafsson SA, von Hausswolff-Juhlin Y, Welch E (2020). Self-admission to inpatient treatment in anorexia nervosa: Impact on healthcare utilization, eating disorder morbidity, and quality of life. Int J Eat Disord.

[14] Strand M, Sjöstrand M (2019). Self-admission in psychiatry: The ethics. Bioethics.

[15] Birgegård A, Björck C, Clinton D (2010). Quality assurance of specialised treatment of eating disorders using large-scale internet-based collection systems: Methods, results and lessons learned from designing the Stepwise database. Eur Eat Disord Rev.

[16] Emilsson L, Lindahl B, Köster M, Lambe M, Ludvigsson JF (2015). Review of 103 Swedish Healthcare Quality Registries. J Intern Med.

[17] Ludvigsson JF, Almqvist C, Bonamy A-KE, Ljung R, Michaëlsson K, Neovius M (2016). Registers of the Swedish total population and their use in medical research. Eur J Epidemiol.

[18] Strand M, Gustafsson SA, Bulik CM, von Hausswolff-Juhlin Y (2017). Self-admission to inpatient treatment in psychiatry: lessons on implementation. BMC Psychiatr.

[19] Cookson R, Mirelman AJ, Griffin S, Asaria M, Dawkins B, Norheim OF (2017). Using Cost-effectiveness analysis to address health equity concerns. Value Heal.

[20] Husereau D, Drummond M, Petrou S, Carswell C, Moher D, Greenberg D (2013). Consolidated Health Economic Evaluation Reporting Standards (CHEERS)—explanation and elaboration: a Report of the ISPOR Health Economic Evaluation Publication Guidelines Good Reporting Practices Task Force. Value Heal.

[21] Strand M, Sjöstrand M, Lindblad A (2020). A palliative care approach in psychiatry: clinical implications. BMC Med Ethics.

[22] Davidson L, Roe D (2007). Recovery from versus recovery in serious mental illness: One strategy for lessening confusion plaguing recovery. J Ment Heal.

[23] Wildes JE, Forbush KT, Hagan KE, Marcus MD, Attia E, Gianini LM (2016). Characterizing severe and enduring anorexia nervosa: An empirical approach. Int J Eat Disord.

[24] Williams KD, Dobney T, Geller J (2010). Setting the eating disorder aside: An alternative model of care. Eur Eat Disord Rev.

[25] Kaplan AS, Miles A, Touyz S, Le Grange D, Lacey JH, Hay P (2016). The Role of Palliative Care in Severe and Enduring Anorexia Nervosa. Managing severe and enduring anorexia nervosa: A Clinician’s Guide.

[26] Russell J, Mulvey B, Bennett H, Donnelly B, Frig E (2019). Harm minimization in severe and enduring anorexia nervosa. Int Rev Psychiatr.

[27] Molin M, von Hausswolff-Juhlin Y, Norring C, Hagberg L, Gustafsson SA (2016). Case management at an outpatient unit for severe and enduring eating disorder patients at Stockholm Centre for Eating Disorders - a study protocol. J Eat Disord.

[28] Dawson L, Rhodes P, Touyz S (2014). The recovery model and anorexia nervosa. Aust New Zeal J Psychiatr.

[29] Ludvigsson JF, Andersson E, Ekbom A, Feychting M, Kim J-L, Reuterwall C (2011). External review and validation of the Swedish national inpatient register. BMC Public Health.

[30] Drummond MF, Sculpher MJ, Claxton K, Stoddart GL, Torrance GW (2015). Using clinical studies as vehicles for economic evaluation. Methods for the Economic Evaluation of Health Care Programmes.

[31] Claxton K (2008). Exploring uncertainty in cost-effectiveness analysis. Pharmacoeconomics.

[32] Arcelus J, Mitchell A, Wales J, Nielsen S (2011). Mortality rates in patients with anorexia nervosa and other eating disorders: A meta-analysis of 36 studies. Arch Gen Psychiatr.

